# Ischemia/Reperfusion Injury in Liver Surgery and Transplantation

**DOI:** 10.1155/2012/453295

**Published:** 2012-12-30

**Authors:** Peter Schemmer, John J. Lemasters, Pierre-Alain Clavien

**Affiliations:** ^1^Sections of Liver Surgery and Transplant Surgery, Department of General, Visceral and Transplant Surgery, University Hospital of Heidelberg, IM Neuenheimer Feld 110, 69120 Heidelberg, Germany; ^2^Center for Cell Death, Injury and Regeneration, Departments of Drug Discovery and Biomedical Sciences and Biochemistry and Molecular Biology, Medical University of South Carolina, USA; ^3^Department of Surgery, University Hospital Zurich, Raemi Street, 8091 Zurich, Switzerland

Despite medical advances, there is unfortunately still no guarantee for adequate liver function after extended resection and transplantation. Impaired liver function is associated with high morbidity and mortality. While the underlying mechanisms are only partly understood, they seem to have similar pathophysiological pathways.

Ischemia/reperfusion injury (IRI) is one of the main contributors to decreased liver function after liver surgery. Posthepatectomy failure is reported in up to 60–90% of cases, despite the fact that the liver remnant's volume in itself should be sufficient to maintain adequate function. After liver transplantation (LT), IRI cannot be avoided and is one of the ultimate factors that limits liver function after LT. Taking into account the various definitions of primary dysfunction and primary nonfunction as well as the number of grafts with high risk of failure and other risk factors, poor graft function is reported in up to 88% of patients after LT. While techniques that do not require hepatic vascular occlusion for liver resection (LR) have evolved, inevitable surgical manipulation itself creates hypoxia to liver tissue during LR, donor hepatectomy, and LT.

Organ specific parameters, such as preexisting damage (i.e., steatosis/steatohepatitis), and additional liver injury, such as surgical trauma, perfusion/preservation solutions, cold/warm ischemia time, and reperfusion, have been identified as contributing towards IRI.

Although some risk factors for mortality and morbidity after hepatic surgery including LT are defined, little is known about the mechanisms of injury. To date, no valid clinical concepts to preserve hepatic integrity and guarantee adequate regeneration in the context of both LT and LR have evolved. Indeed, many protective strategies have been proposed with the aim of preemptively inducing tolerance against IRI or interfering with the pathways of injury and regeneration—either by inhibiting deleterious molecules or enhancing protective pathways. Thus, the focus of this special issue of HPB surgery is on donor preconditioning, warm ischemia, nonheart-beating donors, hemorrhage and resuscitation-related liver injury, antioxidants, liver resection and transplantation, hepatic microperfusion, anesthetic considerations, and small-for-size phenomena.

*Peter Schemmer* *Peter Schemmer* *John J. Lemasters* *John J. Lemasters* *Pierre-Alain Clavien* *Pierre-Alain Clavien*

